# Comparison of Cytomorphometry and Early Cell Response of Human Gingival Fibroblast (HGFs) between Zirconium and New Zirconia-Reinforced Lithium Silicate Ceramics (ZLS)

**DOI:** 10.3390/ijms19092718

**Published:** 2018-09-11

**Authors:** María Rizo-Gorrita, Irene Luna-Oliva, María-Ángeles Serrera-Figallo, José-Luis Gutiérrez-Pérez, Daniel Torres-Lagares

**Affiliations:** Department of Oral Surgery, College of Dentistry, Seville University, Calle de Avicena s/n, 41009 Seville, Spain; marrizgor@alum.us.es (M.R.-G.); irenelunaoliva@gmail.com (I.L.-O.); maserrera@us.es (M.-Á.S.-F.); jlgp@us.es (J.-L.G.-P.)

**Keywords:** zirconia reinforced-lithium silicate ceramic (ZLS), gingival fibroblast, zirconia, computer-aided design/computer-aided manufacturing (CAD/CAM) materials, early cell proliferation, early cell spreading

## Abstract

New zirconia-reinforced lithium silicate ceramics (ZLS) could be a viable alternative to zirconium (Y-TZP) in the manufacture of implantological abutments—especially in aesthetic cases—due to its good mechanical, optical, and biocompatibility properties. Although there are several studies on the ZLS mechanical properties, there are no studies regarding proliferation, spreading, or cytomorphometry. We designed the present study which compares the surface, cellular proliferation, and cellular morphology between Y-TZP (Vita YZ^®^ T [Vita Zahnfabrik (Postfach, Germany)]) and ZLS (Celtra^®^ Duo [Degudent (Hanau-Wolfgang, Germany)]). The surface characterization was performed with energy dispersive spectroscopy (EDS), scanning electron microscopy (SEM), and optical profilometry. Human gingival fibroblasts (HGFs) were subsequently cultured on both materials and early cellular response and cell morphology were compared through nuclear and cytoskeletal measurement parameters using confocal microscopy. The results showed greater proliferation and spreading on the surface of Y-TZP. This could indicate that Y-TZP continues to be a gold standard in terms of transgingival implant material: Nevertheless, more in vitro and in vivo research is necessary to confirm the results obtained in this study.

## 1. Introduction

After the implants’ osseointegration period, second stage surgery requires remodelling the marginal gingival tissues (mainly keratinocytes and fibroblasts) around transgingival abutments [1]. Correct remodelling is essential for direct cellular contact at the level of the implant-abutment interface, which results in a correct mucosal seal [2], avoids apical migration of the junctional epithelium and bone resorption [3], and reduces bacterial adhesion on the surface [4,5].

Furthermore, the material of the transgingival abutment should be biocompatible and able to mimic native tissues as much as possible, providing the best possible substrate to the cells and thus accelerating adhesion, proliferation, and early spreading [1,6,7]. The introduction of computer-aided design/computer-aided manufacturing (CAD/CAM) technology has allowed the use of a wide variety of restorative materials with precise marginal adaptation and reduced processing time. The current gold standard in the manufacture of implant devices is titanium. Nevertheless, according to the latest in vitro studies [7,8,9,10,11], zirconium could be a viable option due to its good mechanical properties, high biological stability, and biocompatibility, as well as lower plaque retention [12,13].

The disadvantage of zirconium is that, due to its white colour and high opacity, it is considered less aesthetic than ceramics [13,14]. The introduction of monolithic zirconium stabilized with yttrium in its translucent (Y-TZP T) and highly translucent versions (Y-TZP HT) for use in CAD/CAM technology has made possible its use as a restorative material without having to be covered by feldspathic ceramics, thus avoiding chipped porcelain and improving its aesthetic properties [15,16].

A systematic review by Linkevicius et al. [17] reported that although there are no statistically significant differences between zirconium and titanium at the bone level, the former is significantly more favourable in terms of the appearance of soft tissues and creates better aesthetics in cases of fine biotype.

Zirconia-reinforced lithium silicate ceramics (ZLS) is a new type of glass ceramic that is considered a valid alternative to lithium disilicate ceramics in cases of high aesthetic demand [18]. ZLS presents a double microstructure composed of very fine crystals of lithium metasilicate and lithium disilicate and a vitreous matrix containing highly dispersed zirconium oxide [14,19,20]. It combines the superior mechanical properties of zirconium—through the transformation from tetragonal to monoclinic phase—with the excellent optical properties of glass ceramics [15,21].

Celtra^®^ Duo (one type of this ceramic) was developed through the manufacturer Degudent (Hanau-Wolfgang, Germany) [22,23]. It is characterized by the inclusion of 10% zirconia in the glass phase, which increases its strength; by the increase of flexural strength from 300.1 MPa after milling to 451.4 MPa after sintered, and an elastic modulus of 70 MPa. Furthermore, it has good optical properties due to a wide range of shades. On the other hand, zirconium is the more studied ceramic. It presents an elastic modulus of 210 MPa and its major advantage is its excellent mechanical strength (800–1200 MPa) [24,25,26,27].

To authors’ knowledge, Celtra Duo has not been compared to Y-TZP in terms of cytotoxicity. Only two studies compared zirconium and Vita Suprinity, presenting both materials adequate values [4,28].

Conversely, there are no studies on early cell proliferation, adhesion, or spreading. We designed the present study which compares the surface, cellular proliferation, and cellular morphology between Y-TZP (Vita YZ^®^ T [Vita Zahnfabrik (Postfach, Germany)]) and ZLS (Celtra^®^ Duo [Degudent (Hanau-Wolfgang, Germany)]) based on nuclear and cytoskeletal measurement parameters in human gingival fibroblasts (HGFs).

The null hypothesis is that there is no difference at the level of proliferation, spreading, or cell morphology between the two materials.

## 2. Results

### 2.1. Surface Characterization

#### 2.1.1. Surface Composition Analysis

**Figure 1:**
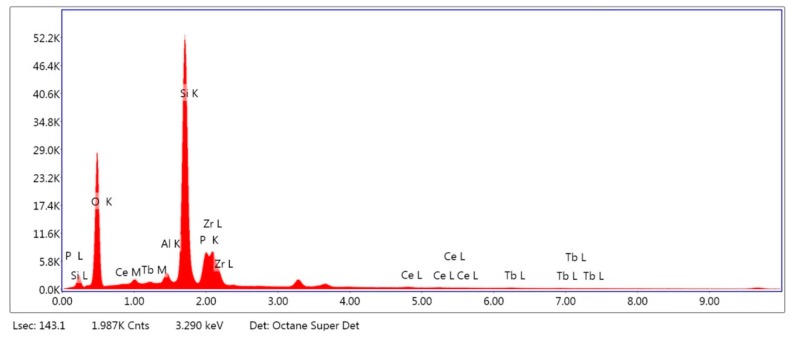
Energy dispersive spectroscopy analysis of Celtra Duo (ZLS).

**Figure 2:**
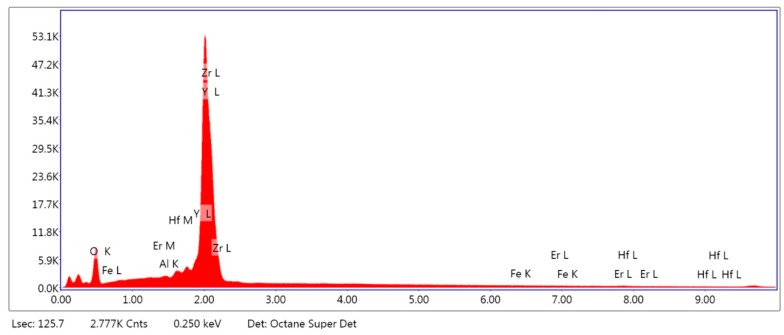
Energy dispersive spectroscopy analysis of zirconium (Y-TZP).

The results from the EDS analysis (Figure 1 and Figure 2) showed high peaks of silicon, oxygen, and potassium in the case of ZLS, and zirconium and yttrium for the case of Y-TZP. Potassium, oxygen, and aluminium were detected in both, while in the case of ZLS, lithium was not detected due to its low molecular weight.

Table 1 shows the percentage of mass content of the chemical compounds detected in each material. Both presented alumina in a reduced percentage. The proportions were similar with those provided by the manufacturer; except in the case of ZLS where the zirconium content was higher than the published value (10%).

#### 2.1.2. Profilometry

**Figure 3:**
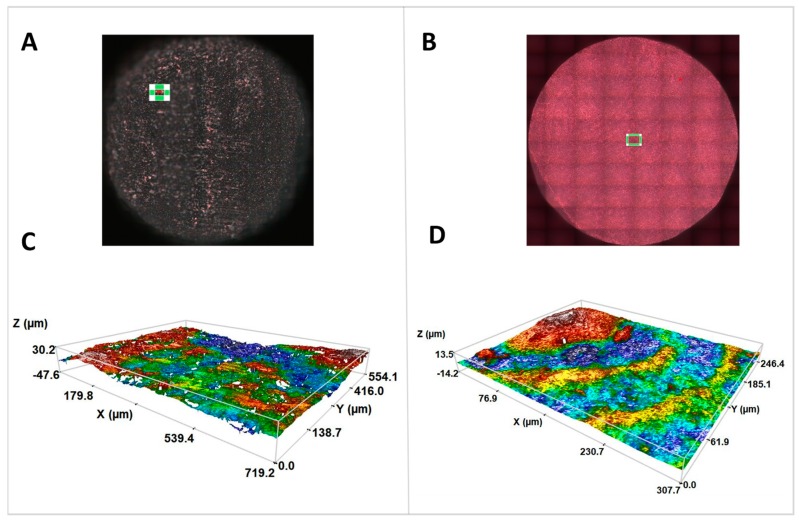
Measurement areas of ZLS (**A**) and Y-TZP (**B**) and profilometry of the samples, objective 20×. (**C**) ZLS; (**D**) Y-TZP.

The measurement areas on the samples and the images obtained from the three-dimensional roughness profile for ZLS and Y-TZP can be seen in Figure 3. A high mean roughness after 5 measurements could be seen in the ZLS sample (Sa (μm) of 2.84 ± 1.14), while Y-TZP surface was shown to be more homogeneous (Sa (μm) of 0.80 ± 0.08).

#### 2.1.3. Surface Topography

**Figure 4:**
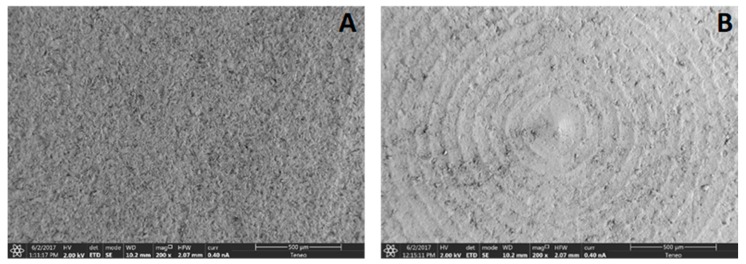
Scanning electron microscopy (SEM) images at 200× magnification of ZLS (**A**) and Y-TZP (**B**).

SEM images of the samples can be seen in Figure 4. ZLS surface revealed a porous, non-homogeneous, irregular structure with crater-like areas and random distribution, while Y-TZP showed concentric and parallel grooves pattern, resulting from the disc manufacturing process. Both surfaces revealed porosities and typical surface defects from the milling and sintering processes.

### 2.2. Cellular Study

#### 2.2.1. Cell Proliferation

**Figure 5:**
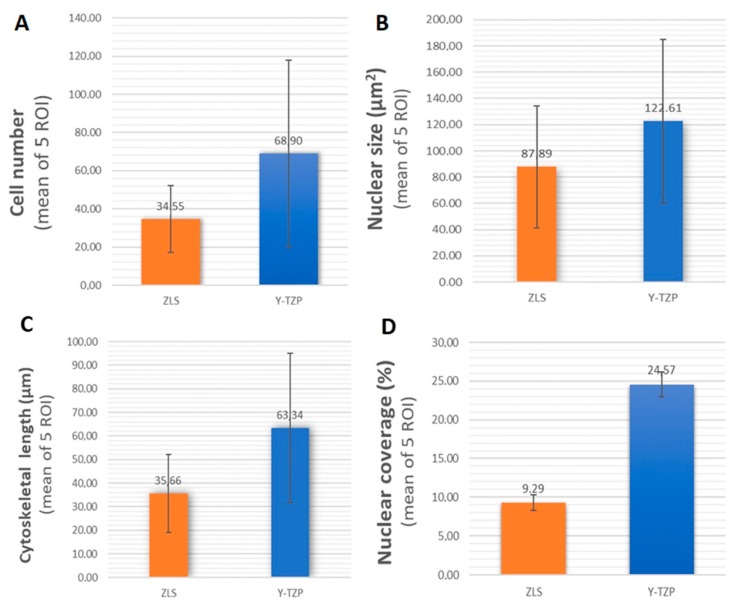
Graphic representations of number of cells (**A**), average size (**B**), length of the cytoskeleton (**C**), and occupied area (**D**).

To assess the surface topography’s effect on the cells, the mean number of cells observed in each image at 20× magnification was quantified. An average of 34.55 ± 17.52 and 68.90 ± 49.02 cells were observed in the case of ZLS and Y-TZP, respectively. According to the U Mann Whitney test, this difference was statistically significant (*p* = 0.039, *r* = −0.371) (Figure 5A).

#### 2.2.2. Cytomorphometry

The mean nuclear size observed for ZLS was 87.89 ± 46.61 μm^2^, while for Y-TZP was 122.61 ± 62.42 μm^2^. According to the U Mann Whitney test, this difference was not statistically significant (*p* = 0.117) (Figure 5B).

Mean length of the main cell axis was 35.66 ± 16.44 μm for ZLS and 63.34 ± 31.64 μm for cells cultured on Y-TZP. According to the U Mann Whitney test, this difference was statistically significant (*p* = 0.000, *r* = −0.468) (Figure 5C).

The mean circularity value of the nuclei was 0.58 ± 0.09 for ZLS and 0.60 ± 0.07 for Y-TZP. According to the *t*-student test, this difference was not statistically significant (*t*_29_ = −0.637, *p* = 0.529).

#### 2.2.3. Nuclear Coverage

Regarding the area occupied by cell nuclei, the percentage observed in ZLS was 9.29 ± 1.03%, while in the case of Y-TZP it was 24.57 ± 1.61%. According to the U Mann Whitney test, this difference was statistically significant (*p* = 0.002, *r* = −0.548) (Figure 5D).

HGFs grown on the surface of ZLS and Y-TZP presented nuclei of similar size and circularity. However, in Y-TZP the number of observed cell and the length of the cytoskeleton and nuclear coverage percentage were higher than those in ZLS.

#### 2.2.4. Morphology and Cellular Attachment

**Figure 6:**
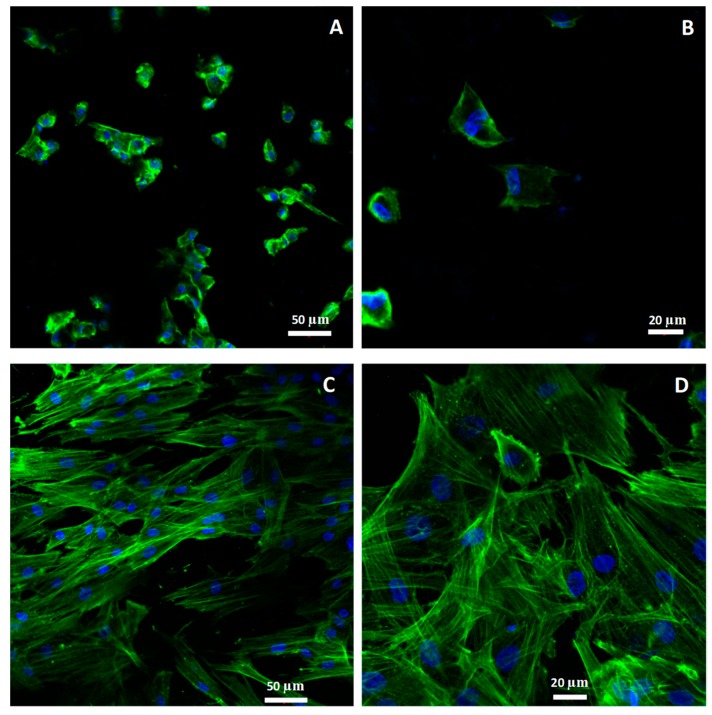
Confocal microscope images of fibroblasts on ZLS at magnification 20× (**A**) and 40× (**B**), and Y-TZP 20× (**C**) and 40× (**D**).

Figure 6 shows the confocal microscopy images of fibroblasts obtained for the observation of cell morphology at magnification 40× and 63×. Double staining was used for the visualization of the nuclei with DAPI (4′,6-diamidino-2-phenylindole, blue) and the actin filaments of the cytoskeleton with phalloidin (green).

In ZLS images, cellular aggregates with weak spreading were observed (Figure 6A). The cell morphology was rounded or quadrangular, with a poor organization of cytoskeletal actin filaments in most cells (Figure 6B), which could be due to a weak initial cell attachment to the disc surface.

In the Y-TZP images, cells with spindle-like morphology are observed in greater numbers, well spread, and uniformly distributed, with a more organized cytoskeleton showing bundles of actin filaments and stress fibers (Figure 6D). These images could be interpreted as a more favourable surface to initial cellular attachment than in ZLS.

These findings are in line with the statistically significant differences found in the number of cells, nuclear coverage, and cell length.

## 3. Discussion

The aim of our study was to evaluate the early cellular response, characterized by proliferation, spreading, covering, and cell morphology of HGFs cultured on Y-TZP and ZLS surfaces.

The results rejected the null hypothesis of equal influence on cellular behaviour. Statistically significant differences were observed in terms of higher mean cell count, cell length, and nuclear coverage on the Y-TZP surface. No significant differences were observed at mean nuclear size, nor at circularity level between both groups.

In this study, statistical differences were evaluated not only based on the *p* value but estimating how they differed, based on effect size tests of the significant variables (Cohen’s d and Normal Approximation *z* to *r*) [29,30]. These tests results showed a moderate effect for both cell proliferation and cell length (*r* = −0.371 and −0.468, respectively) and large effect on nuclear coverage (*r* = −0.548). The cellular morphological analysis was in line with the aforementioned results. According to confocal images, a higher cell count and wider extension of the actin filaments over the Y-TZP surface could be interpreted as a greater cellular affinity after 24 h, the interval at which the images were taken.

The cellular response to the surface of a material depends on multiple variables, including topography, chemical composition of the surface, and phenotype of the cultured cell line [1,7,10].

HGFs are the major cell type in the gingival connective tissue and play a fundamental role in the sealing of soft tissues around the transgingival area of implant abutments [31]. Keratinocytes are another cell line usually studied, given their relevance in the insertion of the junctional epithelium. Several comparative studies of keratinocytes and fibroblasts behaviour seeded on different surfaces have been conducted. In one of them, the keratinocytes viability and migration capacity was negatively influenced on zirconium surfaces of varying composition, while in the case of fibroblasts, they were not affected [1]. Grenade et al. obtained better results in terms of viability, number, and cell coverage when seeding fibroblasts and keratinocytes on titanium and zirconium surfaces compared to lithium disilicate ceramics [32,33]. However, Northdurft et al. cultured epithelial cells and fibroblasts, obtaining different cellular response between both cell lines by growing them on titanium and zirconium surfaces with different surface treatments. Both types of cells had greater proliferation on rough surfaces—a significant finding, since up until that moment a greater proliferation of epithelial cells had been reported on smooth surfaces [7,34].

In our study, human fibroblasts from an immortal cell line were used. It offers greater reproducibility of results than a primary culture, in which cell behaviour can significantly vary from one patient to another [9].

The mean observed cell count had large standard deviations, as in studies where primary cultures were used [32]. In our case, it could be due to the use of cells from increasing passages and not always from the same passage number for each experiment repetition, which could lead to a lower cellular proliferation, as demonstrated by Kent et al. in their study of gingival fibroblasts [35].

The significant difference in three of the five variables (cell count, cell length, and nuclear coverage) could be attributed to the different composition, topography and roughness of both surfaces, as these factors can influence the cell growth and spread, especially in the early stages of proliferation [11].

Material composition is a very influential factor in cell adhesion, with zirconium and titanium observed as having better maintenance of the mucoepithelial barrier than ceramics [36].

In addition to Celtra^®^ Duo [Degudent (Hanau-Wolfgang, Germany)], Vita Suprinity^®^ Vita [Zahnfabrik (Postfach, Germany)] is commercially available. Both are ZLS, but have slight chemical differences: Suprinity^®^ presents potassium oxide (K_2_O: 1–4% wt.) while Celtra^®^ Duo presents terbium oxide (Tb_2_O_3_: 1% wt.), which could influence the cellular response, as reported in the case of zirconium [1]. The advantage of Celtra^®^ Duo (used in this study) is that it can be milled in the final crystallized state, offering the possibility to avoid a second sintering, unless better mechanical properties are preferred [14,19,20,24].

Our results showed mass content (wt %) of zirconium oxide (16.72%) in EDS analysis of ZLS, higher than that announced by the manufacturer (10%) [23]. The peak of lithium oxide, one of the main compounds of this material, was not detected, possibly due to the low molecular weight of lithium [24,25].

As discussed above, factors such as topography or roughness can influence cell morphology, its function, or organization [37]. After sintering process, zirconium presents a less homogeneous surface and a roughness increase due to sintering-induced grain coarsening and residual stresses. In addition, it undergoes a volumetric contraction due to grain growth and material transformation from tetragonal to monoclinic phase [4,26,38].

Our results show that both samples presented an irregular surface, with porosities and surface defects observed in SEM images. However, the Y-TZP surface revealed a concentric and parallel grooves pattern that could have facilitated or guided the attachment and proliferation of the fibroblasts on this surface. Deep grooves and their periodicity influence cell behaviour [39]. Pae et al. [5] cultured fibroblasts in titanium with a smooth surface and zirconium with microgrooves. After twenty-four hours, increased cell proliferation was observed on the surface with micro-roughness. Fibroblasts presented a flattened morphology, widely spread, and oriented following the direction of the microgrooves. Cells cultured on smooth surfaces showed less proliferation and random direction spreading. From these findings, they propose creating microgrooves in the transmucosal implant abutments in order to improve anchorage, allowing for a greater biological seal, an inhibition of epithelial downgrowth, and lower bacterial adhesion. However, some authors disagree. Pabst et al. suggested that smooth surfaces increased cell adhesion and viability and decreased bacterial adhesion [1,4].

In our study, we can see the formation of fibroblast bundles on Y-TZP surface, but not having evaluated the cell culture in a scanning electronic microscope, it is not possible to affirm if these bundles followed the direction of the concentric grooves that characterize the surface of this material.

On surfaces with heterogeneous topography, fibroblasts need more time for initial mechanical stabilization compared to those that grow on more homogeneous surfaces [3,39,40,41]. Cells grown on a rough surface stabilize themselves on topographic irregularities, not needing to develop a strong cytoskeleton. Fibroblasts grown on smoother surfaces in order to mechanically stabilize develop a strong network of actin fibers, appearing more elongated and spread [40,42,43]. This explains why the fibroblasts grown on Y-TZP showed a greater extension of the actin filaments and an elongated shape.

Nothdurft et al. demonstrated that cell area was lower in rougher surfaces. From this finding, it can be deduced that the irregularities of the substrate could have represented a barrier to the extension of the actin filaments of the cytoskeleton on the surface [7,44], as seen on our results.

There is no consensus about which is the best surface to create a good mucosal seal and reduce bacterial adhesion. Some authors have reported the importance of this surface being smooth [8,11,39,42], while other authors have shown that rough surfaces are preferred, either through some surface treatment like sandblasting—with random cell growth—[7,45], or the creation of micro-roughness [5] to enhance attachment, proliferation, and spreading in the direction of the microgrooves.

One factor to be considered is that surfaces with greater roughness have a more hydrophobic behaviour, allowing greater bacterial adhesion on their surface [4].

Fischer et al. and Kournetas et al. postulate that it is necessary to rethink the way in which roughness parameters are applied to in vitro studies. The arithmetic roughness value Ra is a limited factor in complex topographies and is a parameter that depends on experimental conditions and the surface treatment technique employed [42,46].

Caution is advised when comparing our results regarding cellular response based on the value of roughness with other published studies, as in our case the profilometric study was carried out according to ISO 25178, which was not used by other studies.

The stiffness of the substrate can influence cell behaviour more than chemical composition or surface hydrophilicity [40,43,47]. The ability to perceive and respond to the stiffness of the substrate has been observed in fibroblasts, which prefer rigid surfaces, a phenomenon known as durotaxis [48,49]. They have the ability to adapt to the substrate by reorganizing the structure of the actin stress filaments of the cytoskeleton, thus adjusting their intracellular stiffness [50]. In studies of fibroblasts cultured on surfaces of increasing stiffness, it was observed that the greater the increase in the modulus of elasticity of the substrate, the greater the proliferation, area, length, and cell density, allowing a better cell to cell contact, which stimulates the elongation and development of the stress fibers of the cytoskeleton, resulting in a spindle-like phenotype [47,48,49,51,52,53].

The results of our study are consistent with the aforementioned studies. Y-TZP had a modulus of elasticity (210 GPa) [54] more than ZLS (70 GPa) [55,56], which is reflected in the greater proliferation, cell length, and nuclear coverage on Y-TZP surface.

To the authors’ knowledge, there is no comparative studies of cell proliferation, spreading, and morphology of cell cultures on Y-TZP and ZLS surfaces, the present work being the first study in this matter. However, two studies about cellular viability and cytotoxicity have been performed in which Suprinity^®^ is compared with Y-TZP. In one, cell viability rate was not statistically significant at 24 h when comparing Suprinity^®^ with zirconium. However, the initial apoptosis rate was significantly higher in the zirconium [28]. In the other study, cell viability was studied at 24 h in two surface treatments. A higher cell viability was observed in zirconium for both polished and glazed surfaces [4].

Conversely, early cellular response in zirconium has been compared with multiple restoration materials, such as feldspathic ceramics [9,10] or titanium [7,8]. In vitro, zirconium is considered the alternative to titanium in terms of better maintenance of soft tissue stability, since it provides better biocompatibility and aesthetics [1,9,31].

Raffaeli et al. made a comparative study of zirconium and feldspathic ceramics, finding higher cell growth rate in the zirconium at the first 24 h [9].

As cell shape is a good indicator of the physical effects of a substrate on cellular behaviour regarding proliferation, adhesion, or spreading [57], we conducted a cytomorphometric study based on nucleus and cytoskeleton measurement parameters.

It has been observed that cell spreading and nuclear geometry are closely related [58,59]. In our study, no statistically significant differences were observed in the size or nuclear circularity between ZLS and Y-TZP.

Another indicator of cellular response is the cytoskeleton shape, which is a regulatory structure of the signals perceived from the environment and coordinates cell size, shape, and function [60]. After initial cell attachment to a surface, spreading, proliferation, and cell migration take place [61].

The average cell length observed in our study for Y-TZP surface (63.34 ± 31.64 μm) is in line with that published by other authors for an extended fibroblast [48]. However, in the case of ZLS (35.66 ± 16.44 μm), the average length was approximately half of that of Y-TZP. These results are in line with those of the cytomorphometric analysis, since on Y-TZP surface, extended cells with spindle-like phenotype were observed with a more organized cytoskeleton in actin filaments bundles and stress fibers. Cells cultured on ZLS showed cellular aggregates, a rounded phenotype with a more cortical cytoskeleton and weaker extension of the actin filaments. From these results, ZLS can be inferred as a less favourable surface for early attachment and proliferation.

Our results showed greater nuclear coverage on Y-TZP. Cellular coverage percentage is a variable that can be calculated from the value of the area occupied by the nuclei multiplied by the value of the nucleocytoplasmic ratio (N:C), which in the case of the fibroblast is 0.306 [62]. This ratio is generally used in cell biology to measure cell size [63].

The resulting formula would be the following: $\%\;nuclear\;area\times \frac{nuclear\;area}{cell\;total\;area}$. Therefore, cellular coverage area in ZLS is $\frac{9.29}{0.306}$ = 30.35%, and in the case of Y-TZP it is $\frac{24.57}{0.306}$ = 80.29%.

Several studies have shown that cell morphology is associated with spreading, adhesion, and cell division. Flattened and more spread out cells have a higher division rate and more favourable adhesion than circular ones with scarce spreading. This reasoning is in line with the results obtained in our study. Cells cultured on Y-TZP resulted in more coverage of the disc surface [5,64,65].

As mentioned above, there are several factors that influence initial cellular adhesion to a surface, such as chemical composition, mechanical properties (such as wettability or stiffness), or the cytotoxicity of the material. Therefore, this is a multiparametric and complex analysis, in which each parameter should be studied independently under an ISO standard, so that in vitro studies could be reproducible with objectively comparable results [32,42,46]. Some examples are ISO 10993:2009 (Part 5 based on vitro cytotoxicity tests, Part 12 based on sample preparation, and Part 19 based on physico-chemical, morphological, and topographical characterization of materials). 

## 4. Materials and Methods

### 4.1. Sample Preparation

Two CAD/CAM materials were studied for all-ceramic restorations: VITA YZ^®^ T [Vita Zahnfabrik (Postfach, Germany)] and Celtra^®^ Duo [Degudent (Hanau-Wolfgang, Germany)]. Its composition, manufacturer’s data, and reference are shown in Table 2.

From Y-TZP discs and ZLS blocks used for CAD/CAM ceramic restorations, disc-shaped samples (size 10 mm × 2 mm) were milled. The Y-TZP samples were sintered according to the manufacturer’s recommendations. ZLS commercial blocks are fully crystallized, being only necessary to mill the block in a disc form.

Discs were then cleaned by immersion with absolute ethanol and sterilized with UV exposure for 30 min on each side inside laminar flow workstation. Finally, discs were place on its base in a sterile Petri dish before performing the experiment.

### 4.2. Surface Characterization

Prior to the experiments, composition, roughness profile, and surface morphology of each of the discs were analysed.

#### 4.2.1. Surface Composition Analysis

Surface composition analysis was performed by energy dispersive spectroscopy analysis (EDS) using a scanning electron microscope (FE-SEM) FEI TENEO (Thermo Fisher Scientific Inc., Waltham, MA, USA) with field emission gun Schottky type and EDAX METEK SDD detector. EDAX TEAM software version 4.4.1 was used for image processing. An area of 130 μm was analysed for 200 s. The results of the microanalysis are expressed as a percentage of mass content (wt %).

#### 4.2.2. Profilometry

The roughness study was performed using the confocal-interferometric microscope Sensofar S NEOX (Sensofar Medical, Terrassa, Spain). The software used was SensoMAP Premium 7.4. The measurements were made in accordance with ISO 25178: Geometric Product Specifications (GPS)—Surface texture: Areal. A 20× epi-illumination lens was used at a focal length of 4.50 mm and a green optical resolution of 0.32 μm. Five measurements were made randomly and in different locations for each material, with a pre-established dimension of 0.87 × 0.66 mm^2^ and a cut-off correction of 250 μm. The quantitative roughness parameter used was the arithmetic mean and the standard deviation of the 3D roughness (S_a_).

#### 4.2.3. Scanning Electron Microscopy

The FE-SEM analysis of the surface morphology was performed with the FEI TENEO scanning electron microscope (SEM) (Thermo Fisher Scientific Inc., Waltham, MA, USA) at 200× magnification.

### 4.3. Cell Culture

Human gingival fibroblasts (HGF, Lonza, Basel, Switzerland) were cultured in T75 flasks in an incubator (5% CO_2_, 95% air at 37 °C). The culture medium prepared was Dulbecco’s Modified Eagle Medium (DMEM, Biowest, Nuaillé, France) supplemented with 10% fetal bovine serum (FBS, Biowest) and 1% glutamine-penicillin-streptomycin (Biowest, Nuaillé, France) for each 500 mL of culture medium.

The medium was changed every 48 h and the cells were subcultured regularly upon reaching 80% confluence using Dulbecco’s phosphate-buffered saline without calcium and magnesium (DPBS, Lonza, Basel, Switzerland) and 0.25% trypsin heated at 37° (Biowest, Nuaillé, France).

For control and cell counting while culturing in T75 flasks, an inverted phase contrast microscope Olympus CKX41SF2 (Olympus, Shinjuku-ku, Tokyo, Japan) was employed. The initial adhesion, growth, and cellular spread between the passages were verified. Cells were seeded on the discs from the 3rd passage and never reached 10th passage, as indicated by the manufacturer.

Samples of both materials were glued on its base to a sterile Petri dish. HGFs were grown on the discs at a previously calculated concentration of 1 × 10^3^ cells in 40 μL of culture medium per disc. After several hours, the initial cellular adhesion was checked on the disc surface with an Olympus CKX41SF2 inverted phase contrast microscope (Olympus, Shinjuku-ku, Tokyo, Japan). Subsequently, the Petri dish was filled with culture medium up to the level of the discs’ surface using a pipette. Cells were then incubated for 24 h before immunocytochemical staining.

#### 4.3.1. Immunocitochemical Staining

After 24 h of cell culture in the discs, the medium was removed with a pipette. After washing with 500 μL DPBS twice, it was fixed with 4% paraformaldehyde in DPBS (AppliChem GmbH, Darmstadt, Germany) for 10 min, then washed with DPBS before permeabilizing with 0.1% Triton X-100 (Sigma, Saint Louis, MO, USA) at 4 °C for 5 min. Samples were washed again, and the blocking phase of nonspecific background staining was performed by applying 1% bovine serum albumin (BSA) in PBS for 20 min. After removal of BSA excess with a pipette, immunohistochemical staining was performed with 15 μL of Acti-stain 488 Fluorescent Phalloidin (Cytoskeleton, Inc., Denver, CO, USA) per disc. This staining is a mycotoxin that has high affinity for F-actin, present in the filaments of the cellular cytoskeleton. For DNA staining of the cell nuclei, 15 μL of 4′,6-diamidino-2-phenylindole (DAPI) was also used, which is incorporated in the Vectashield mounting medium (Vector Laboratories Inc., Burlingame, CA, USA). Phalloinide stain was applied and left for 30 min in the dark before washing again with DPBS and applying the DAPI with the Vectashield mounting medium and the coverslip. The samples were kept at 4 °C and observed under the confocal microscope after 24 h.

#### 4.3.2. Confocal Microscopy

The samples were observed with a Zeiss LSM 7 DUO confocal microscope (Carl Zeiss, Jena, Germany) with an Excite 120PC epifluorescence unit. Images were obtained using the FITC at 488 nm laser and fluorescence emission was collected through 535 nm band pass filter for the cytoskeleton visualization; for the visualization of the nuclei, the excitation filter DAPI at 355 nm was used and collected the emission through 458 nm band pass filter, all in accordance with the parameters recommended by the manufacturer. Image analysis software “ZenLite” 2012 was used (Carl Zeiss, Jena, Germany), along with three Plan-Apochromat objectives of 20×/0.8, 40×/1.30 Oil DIC and 63×/1.40 Oil DIC. Images were taken in 5 regions of interest (ROI) per disc (northwest, northeast, centre, southwest and southeast). No sample was exposed to the laser for more than 5 min in order to avoid photobleaching.

#### 4.3.3. Image Processing: Image J

Images of 2048 × 2048 pixels were obtained at 8-bit, which were saved in tiff format for later analysis with the ImageJ v1.50e program (Wayne Rasband, National Institutes of Health, Bethesda, MD, USA). With the 20× objective images, the number of cells and the disc coverage were analysed; with the 40× and 63×, cell morphology was analysed and cell size measurements were made.

Each image was calibrated to μm before processing. For cell count, the RGB channels (red, green, and blue) were separated from the original image at 20×. With the “blue” image, an automatic threshold was established which identified the cell nuclei from the creation of a binary image (*Image* → *Adjust* → *Threshold* → *Auto*). The *Process* → *Binary* → *Watershed* command was applied to automatically separating or cutting apart nuclei that touched each other. Finally, the *Analyze* → *Analyze Particles* command was applied, in which the minimum size of a nucleus to be detected was set at 5 μm^2^.

In the case of cell length measurement, after image calibration at μm, a linear selection of the main axis of the cytoskeleton of 3 cells chosen randomly in each 40×-oil photo was made. The variable length is represented as the length in μm.

#### 4.3.4. Cellular Parameters Analyzed

The variables analysed were the number of cells (proliferation), the average size of the nucleus (*nuclear size*), its circularity (*circularity*), the percentage of area occupied by the nuclei with respect to the total image (*% nuclear coverage*), and average length of the main axis of the cytoskeleton (*cell length*).

The circularity value of the nucleus oscillates between 0 and 1 [66]. Values closer to 0 are interpreted as elongated forms; on the other hand, the value of 1 is interpreted as a perfect circle. It is based on the following formula: $\frac{{\left(\mathrm{Perimeter}\right)}^{2}}{4\pi Area},$ perimeter = 2π*r*.

The cellular parameters analysed—which can be observed within the Results section—are classified as cell proliferation, cytomorphometry, and nuclear coverage. Cell morphology and attachment are based on the visualization of the SEM images. All parameters are expressed in values of mean ± standard deviation for the images observed in each of the variables.

### 4.4. Statistic Analysis

The comparison of the two groups for each of the analysed variables was made using IBM SPSS Statistics 24.0 software (International Business Machines Corp, New York, NY, USA). The Kolmogorov-Smirnov test was performed to verify the existence of normality. For those variables with normal distribution, the Levene test was employed to check the variance homogeneity prior to the application of t-student. For the variables in which normality was not verified, the U Mann Whitney test was applied. A level of statistical significance of 5% was established (*p* < 0.05). In the cases of variables with a normal distribution in which statistically significant differences were identified, the magnitude of the effect was calculated through Cohen’s d. Scale interpretation for the values of Cohen’s d is based on standard deviation units: 0.2 (small effect), 0.5 (moderate effect), and 0.80 (large effect) [29]. Normal Approximation *z* to *r* (based on the formula *r* = $\frac{Z}{\sqrt{N}}$, where *N* is total number of scores) was used for those variables with non-normal distribution. Scale interpretation was valued on Cohen’s criteria of 0.1 (small effect), 0.3 (moderate effect), and 0.5 (large effect) [30].

## 5. Conclusions

The results of this study suggest that HGFs cultured on Y-TZP have a greater cell proliferation, coverage, and spreading than those cultured on ZLS, which translates in a greater affinity for the surface of Y-TZP.

Several factors influence the initial cellular adhesion to a surface, and although not all have been analysed in the present study, the better cellular response in Y-TZP could be attributed to a more homogeneous and less rough surface topography. Hence Y-TZP can still be considered a *gold standard* even when compared against new ceramic materials such as ZLS.

In order to establish comparable results, future studies should be reproducible under ISO standards.

In future studies, rugosity parameters should be homogenised as it can be a confounding variable during the period of early cellular response.

Due to the limitations of this study, more in vitro and in vivo studies are necessary to verify the obtained results.

## Figures and Tables

**Table 1:** Mass content (%) of Celtra Duo (ZLS) and zirconium (Y-TZP) by energy dispersive spectroscopy (EDS) analysis.

Material	CELTRA DUO (ZLS) [wt %]	Material	Y-TZP [wt %]
Al_2_O_3_	2.85	Al_2_O_3_	0.08
SiO_2_	71.01	ZrO_2_	88.66
P_2_O_5_	7.44	Y_2_O_3_	10.41
ZrO_2_	16.72	Fe_2_O_3_	0.16
Ce_2_O_3_	1.10	Er_2_O_3_	0.26
Tb_2_O_3_	0.89	Hf_2_O_3_	0.43

**Table 2:** Summary of the characteristics of the studied materials.

Name Material Type Manufacturer Ref./Lot Number			
VITA YZ^®^ (Y-TZP)	Zirconium dioxide partially stabilized with yttrium oxide	VITA Zahnfabrik, Bad Säckinger, Germany	YZ T^white^/63320
Celtra^®^ Duo (ZLS)	Lithium silicate vitreous ceramic reinforced with zirconium	Degudent GmbH, Hanau-Wolfgang, Germany	HT-A1/18027341

## Abbreviations

CAD/CAM	Computer Aided Design/Computer Aided Manufacturing
Y-TZP	Yttria-stabilized Tetragonal Zirconia Polycrystal
HGFs	Human Gingival Fibroblasts
ZLS	Zirconia Lithium Silicate
EDS	Energy Dispersive Spectroscopy
SEM	Scanning Electron Microscopy
